# Training strategies of 10,074 athletes from 121 countries based on human development index in early COVID-19 lockdown

**DOI:** 10.1038/s41598-024-59375-y

**Published:** 2024-04-17

**Authors:** Olivier Galy, Jad Adrian Washif, Guillaume Wattelez, Abdulaziz Farooq, Olivier Hue, Øyvind Sandbakk, Christopher Martyn Beaven, Stephen Seiler, Ding Ding, David B. Pyne, Karim Chamari

**Affiliations:** 1 Interdisciplinary Laboratory for Research in Education, University of New Caledonia, Nouméa, New Caledonia; 2 Sports Performance Division, Institut Sukan Negara Malaysia (National Sports Institute of Malaysia), Kuala Lumpur, Malaysia; 3 Aspetar, Orthopaedic and Sports Medicine Hospital, Doha, Qatar; 4 Laboratoire ACTES, Université des Antilles, Pointe-À-Pitre, Guadeloupe France; 5 Centre for Elite Sports Research, Department of Neuromedicine and Movement Science, Norwegian University of Science and Technology, Trondheim, Norway; 6 Te Huataki Waiora School of Health, University of Waikato, Tauranga, New Zealand; 7 Department of Sport Science and Physical Education, Faculty of Health and Sports Sciences, University of Agder, Kristiansand, Norway; 8 Sydney School of Public Health, Faculty of Medicine and Health, The University of Sydney, Camperdown, NSW Australia; 9 Charles Perkins Centre, The University of Sydney, Camperdown, NSW Australia; 10 Research Institute for Sport and Exercise, University of Canberra, Canberra, Australia; 11 Higher Institute of Sport and Physical Education, ISSEP Ksar Saïd, Manouba University, Manouba, Tunisia; 12 Naufar Wellness and Recovery Center, Naufar Wellness and Recovery Center, Doha, Qatar

**Keywords:** Human development index, Health, Socio-economic inequity, Physical activity, Education, Sport, Health care, Medical research

## Abstract

The aim of this study was to investigate relationships between changes in training practices and human development index (HDI) levels, and identify strategies employed by athletes who consistently maintained their training quantity during the first 100 days of the COVID-19 pandemic. A total of 10,074 athletes (5290 amateur and 4787 professional athletes from 121 countries) completed an online survey between 17 May to 5 July 2020. We explored their training practices, including specific questions on training frequency, duration and quantity before and during lockdown (March–June 2020), stratified according to the human development index (HDI): low-medium, high, or very high HDI. During the COVID-19 lockdown, athletes in low-medium HDI countries focused on innovative training. Nevertheless, women and amateur athletes experienced a substantial reduction in training activity. Performance-driven athletes and athletes from higher HDI indexed countries, were likely to have more opportunities to diversify training activities during lockdowns, facilitated by the flexibility to perform training away from home. Factors such as lockdown rules, socioeconomic environment, and training education limited training diversification and approaches, particularly in low-medium and high HDI countries. Athletes (amateurs and professionals) who maintained the quantity of training during lockdown appeared to prioritize basic cardiovascular and strength training, irrespective of HDI level. Modifying training and fitness programs may help mitigate the decrease in training activities during lockdowns. Customized training prescriptions based on gender, performance, and HDI level will assist individuals to effectively perform and maintain training activities during lockdowns, or other challenging (lockdown-like) situations.

## Introduction

The first 100 days of the lockdown initiated at the beginning of the COVID-19 pandemic resulted in the closure of “non-essential” businesses (e.g., gyms, outdoor sports amenities, playgrounds) which negatively affected people’s daily lives including dramatic decreases in training practices^1,2^. In the early months of the pandemic, exercise levels declined substantially^3,4^, but later a compensatory “rebound” was reported^5^. Interestingly, analysis of Google™ searches revealed a surge in community interest in ‘home-based’ exercises like high-intensity interval training^6^, suggesting a societal interest to adapt training during lockdowns^7^. There was a shift towards digitally delivered coaching/training during lockdowns^8^.

During the lockdown periods, the levels of modification in training routines were largely depended on sports, with minimal variation between sexes^8,9^. Even top-level athletes, who had the advantage of specialised equipment and technology access, experienced considerable challenges in maintaining their training during lockdowns^8,9^. The human development index (HDI) can be useful to obtain a global understanding on how athletes at various levels, and countries, were affected by lockdown during the COVID-19 pandemic. Indeed, the HDI is a multi-dimensional indicator of socioeconomic development that considers factors such as life expectancy, education, and income. Considering that during the global health crisis, countries with more overstretched health care system tend to be highly burdened^2,10, 11^, the HDI and its components, may potentially explain discrepancies in the global training practices^12^.

Contextualization of training data with HDI could explain variations in training practices in countries with different levels of HDI during lockdowns. Therefore, the aim of this study was twofold: (i) to investigate the relationship between changes in training parctices and HDI levels; (ii) to identify the strategies employed by athletes who best maintained quantity of training during the first 100 days of the COVID-19 pandemic. We hypothesized that by linking geographical and temporal patterns of training practices and athlete strategies to corresponding levels of social and economic progress (HDI), we would identify insights to inform sports and public health policies during challenging situations such as the early COVID-19 lockdown.

## Materials and methods

### Study design

This cross-sectional study is based on data from the ECBATA consortium (Effects of Confinement on knowledge, Beliefs/Attitudes, and Training in Athletes consortium)^8,9^. The sample size estimation for this study was 9461 (2% margin of error, and 99.99% confidence level), based on factors of the world’s population size, and normal distribution of sample (http://www.raosoft.com/).

### Participants

Participants provided informed consent, and the study received the ethical approval from the University of Melbourne (HREC No. 2056955). This research was conducted in accordance with the principles outlined in the Declaration of Helsinki. Furthermore, the principles of the General Data Protection Regulation (gdpr-info.eu) were followed to preserve confidentiality of participants during the data collection. All participants were informed of their freedom to withdraw or rescind their agreement to the data collection at will.

The eligibility criteria were: (i) individuals aged ≥ 18-years-old with or without a disability; (ii) had experienced ≥ 2 consecutive weeks of the early lockdown (March to June 2020); (iii) had not missed training for ≥ 7 days due to illness and/or injury during the survey period; and (iv) experienced a ‘medium to high’ lockdown severity level, defined as meeting one or more of the following conditions: (a) movement was limited for essential supplies and groceries only; (b) access limitations to public exercise facilities were imposed (i.e., limited access, or closure of recreational places such as parks or open spaces were not allowed and/or time/capacity limited); and (c) closure of public and private training facilities, at colleges, clubs, institutions. Responses from 10,074 athletes from 18 to 76 years old (self-described as 5290 amateur and 4787 professional and semi professional athletes from 121 countries) were included in statistical analyses.

### Measures

#### Data collection

Google Forms was used to administer and distribute the survey between May and July 2020. The survey was circulated among the research team’s professional networks via personal/group messaging applications (e.g., WhatsApp, Signal, Telegram, etc.), e-mail and further promoted on social media (e.g., Facebook, Instagram, and Twitter). The survey was translated and administered in 34 languages. The research team, which comprised at least one native speaker and one topic expert as native speakers of each of these 34 languages, translated and back-translated the survey questions, as well as pilot survey completions. Final surveys for all languages resulted from native language speaking participants’ feedback.

Using an automatic/customized function on the excel spreadsheet (Microsoft Corporation, Redmond, WA, USA), data from questions with pre-determined answers (i.e., pre-defined multiple choice) were directly converted into standardized codes/numbers. All automated responses were checked. Remaining data (i.e., free-text responses) were submitted to theme analysis and/or aggregation (all non-English responses were first back-translated to English). Thereafter, subsequent themes were re-classified into standardized codes/numbers enabling statistical analysis. The test–retest reliability of the survey instrument (9 ± 4 days apart) was determined within a sub-group of English-speaking participants and rated as good to excellent (ICCs 0.82 to 0.97). More detailed procedures of the data collection are described elsewhere^8,9^.

#### Survey questionnaire

The primary group of ECBATA consortium designed the survey, which was then reviewed by the research team of over 100 academics and sports scientists from 60 different countries out of the ECBATA consortium’s full survey. We extracted data from 18 questions for this analysis, divided into sections:

- Participant details (7 questions): participant characteristics including sex, age, years of regular exercise/training, household size, personal economy and participants context including the duration of the activities allowed during the lockdown, and athlete classification (amateur, professional).
- Training practices (11 questions): a mix of question styles was used to establish training practices, including: (i) binary yes or no; (ii) comparing related pre- to during-lockdown effects on training practices (i.e.: frequency and duration); (iii) selecting one or more or predefined answers; and (iv) a free-text cell allowing self-reporting of more nuanced details.

Based on frequency of training (times per week) and duration (minutes per week) before and during lockdown, we calculated the quantity of training per week (quantity of training = frequency × duration) for the same periods. We considered athletes who reach current international physical activity recommendations, defined as adults undertaking “150–300 min of moderate-intensity, or 75–150 min of vigorous-intensity physical activity, or some equivalent combination of moderate-intensity and vigorous-intensity aerobic physical activity, per week”^13^. While physical activity encompasses all daily activities, in this study we focused on a critical element of this physical activity: training practices that correspond to moderate- or vigourous- intensity activities.

#### Human Development Index

The HDI was introduced in 2019 by the UN Development Programme (UNDP)^14^. HDI comprises three dimensions of human development: (i) a long and healthy life (based on life expectancy at birth); (ii) access to knowledge (based on a combination of adult literacy rate and primary education to tertiary education enrolment rates); (iii) and a decent standard of living (based on GDP per head adjusted for purchasing-power parity [US$]). We used categorised HDI in four categories as per the UN Development Programme (UNDP): *low* (HDI < 0.5), *medium* (0.5 ≤ HDI < 0.8), *high* (0.8 ≤ HDI < 0.9), and *very high* (HDI ≥ 0.9)^14^.

#### Statistical analysis

All data were processed with statistical analyses performed using R software version 4.1.0^15^. Alpha was set at α = 0.05. Data are presented using a variety of appropriate descriptive statistics, including percentages, and mean (standard deviation). Significance of relations between factors and HDI levels were tested through χ^2^ test procedure for comparing proportions across categorical variables, and one-way analysis of variance (ANOVA) procedure used to compare means between categorical variables with more than two levels. A posthoc Bonferroni correction was performed to adjust for multiple comparisons. Given a limited number of participants from countries in the *low* HDI category, we subsequently merged the *low* and *medium* categories to form a “*low-medium* HDI”. When comparing numeric factors according to both time (i.e. before and during lockdown) and HDI levels, we performed general linear model univariate ANOVA with (HDI level: *low-medium* vs. *high* vs. *very high*) as between factors × (time: before lockdown vs. during lockdown) as within factor. Additional to this when including the participants’ status in the analysis, a three way ANOVA 3 × 2 × 2 (status: amateur vs. professional) ANOVA procedure was performed. Logistic regression analysis was performed to with dependent variable training quantity maintenance was introduced to characterize whether participants maintained and/or increased their training quantity during lockdown. Data were processed with a predictive approach according to HDI levels which permitted us to determine the main factors explaining training quantity maintenance. For the predictive analysis (SM1), we divided participants in two groups, those who maintained their PA were tagged as “YES” and those who did not maintain their PA were tagged as “NO”. More details about factor selection procedures are provided in supplemental material (SM1)^16^.

## Results

### Sociodemographic characteristics and lockdown description

**Figure 1:**
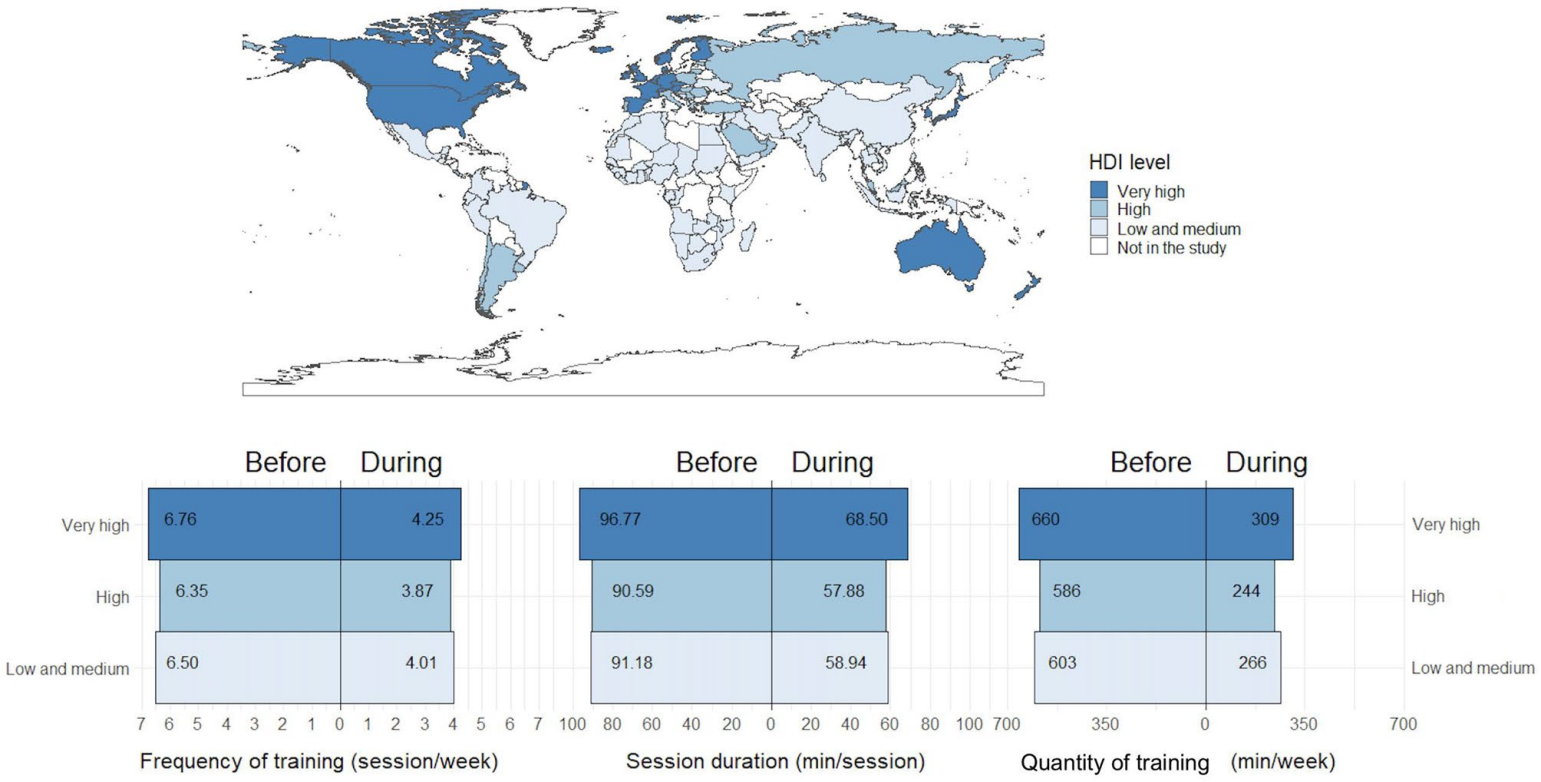
Human development index (HDI) of the 121 countries. Amateur (n = 5290) and professional athletes (n = 4787) participated in this study. HDI values observed were between 0.394 to 0.957 according to Human Development Data Center and Global data laboratory^14^. Participant numbers were *low/middle* (n = 4193), *high* (n = 2651), and *very high* HDI (n = 3230). This figure presents practices of the participants before and during lockdown through frequency of training, session duration and quantity of exercise (Frequency × Duration) in the *low-medium*, *high* and *very high* HDI level countries.

Before lockdowns, athletes reported engaging in at least 150 min of MVPA per week (Fig. 1). Meanwhile, the years of practice, duration of lockdown, and household size of amateurs and professional athletes are comparable, as shown in Table 1.

**Table 1:** Socio-demographic characteristics, authority rules, general purpose of training, financial issues in amateur and professional athletes during the first 100 days of the COVID-19 pandemic according to the HDI level.

		Amateur group								Professional group							
		Female				Male				Female				Male			
		Low and med	High	Very high	p-value	Low and med	High	Very high	p-value	Low and Med	High	Very high	p-value	Low and Med	High	Very high	p-value
	Size, n	720	452	622		1220	877	1399		776	412	453		1477	910	756	
Participants	Age, year (mean (sd))	26.9 (10.3)	27.1 (8.9)	27.1 (9.7)	0.892	29.2 (11.1)	31.5 (11.1)	29.7 (12.1)	< 0.001	24.6 (7.2)	24 (5.8)	25.6 (6.8)	0.002	26.9 (8.4)	26.4 (7.1)	29.2 (10.7)	< 0.001
	Years of practicing (mean (sd))	7.9 (7)	9.6 (7.4)	11 (7.5)	< 0.001	9.8 (8.4)	11.3 (8.7)	12.7 (8.1)	< 0.001	9.9 (5.3)	10.8 (6)	11.7 (5.8)	< 0.001	11.6 (7.1)	12.5 (6.3)	12.9 (6.2)	< 0.001
	Duration of lockdown, week (mean (sd))	9.4 (3.1)	9 (3)	8.5 (2.5)	< 0.001	8.8 (3.0)	8.7 (3)	7.8 (2.7)	< 0.001	8.9 (3.7)	8.2 (3.2)	8.1 (3.0)	< 0.001	8.3 (3.6)	8.2 (3.3)	8.1 (2.9)	0.504
	Household size, n (mean (sd))	3.7 (1.2)	3.5 (1.2)	3.1 (1.3)	< 0.001	3.6 (1.1)	3.5 (1.2)	3.4 (1.2)	< 0.001	3.7 (1.2)	3.6 (1.2)	3.1 (1.3)	< 0.001	3.7 (1.1)	3.6 (1.2)	3.2 (1.2)	< 0.001
While in lockdown, the local authorities allowed	Exercising at home only (%)	77.2	67.8	51.8	< 0.001	73.2	64.9	51.7	< 0.001	74.4	72.2	62.8	< 0.001	68	73.7	62.0	< 0.001
	Receive/borrow equipment (%)	15.1	18.9	20.9	0.018	13.5	13.1	14.3	0.703	19.5	24.1	24.6	0.057	15.8	21.7	24.4	< 0.001
	Using available spaces for exercise around housing area (%)	42.04	48.8	45.8	0.065	39	45.3	41.5	0.016	41.2	49.7	45.5	0.017	40.3	49.2	40.3	< 0.001
	Running in a recreational park or stadium (%)	21.4	21.8	44.4	< 0.001	24.8	21.3	41.7	< 0.001	21	19.7	43.5	< 0.001	23.1	23.4	34.4	< 0.001
	Outdoor cycling (%)	22.2	32.8	56.2	< 0.001	25	26.1	40.9	< 0.001	21.8	20	49.7	< 0.001	16	19.4	36.9	< 0.001
	Access to gymnasium (%)	1.9	0.6	3.8	0.002	2.9	1.7	4.7	< 0.001	6	2.9	12.2	< 0.001	6.9	4.1	8.5	0.001
	Access to sports academy (%)	1.2	1.1	3.7	0.002	2.2	0.4	7.9	< 0.001	7.3	1.2	7.7	< 0.001	5	2.3	6.4	< 0.001
	Outdoor hiking or trekking in non-public facilities (%)	17.7	27.9	44.9	< 0.001	16.8	26.1	24	< 0.001	11.3	16.1	38.2	< 0.001	14.5	20.9	24.9	< 0.001
	Other (%)	0.8	1.1	1.4	0.571	0.4	1	0.8	0.360	1.2	0.2	1.1	0.207	0.6	0.4	1.6	0.016
General purpose(s) of training during lockdown	General fitness and health (%)	86.6	88.6	83.6	0.059	81.7	83.7	80.1	0.098	84.7	84.2	88.2	0.158	78.3	85.9	84.2	< 0.001
	Skills/technique (%)	44.1	38.8	38.9	0.094	40.4	35	38.1	0.042	57.2	43.6	46	< 0.001	47.1	47.3	42.8	0.108
	Strength and power (%)	53	52	51.3	0.835	51.3	50.7	54.7	0.098	61	56.8	61.7	0.265	53.6	65.7	59.9	< 0.001
	Muscular endurance (%)	56.9	60.2	54.4	0.170	51.5	51.1	54.7	0.152	60.7	59.4	61.5	0.825	52	64.6	59.8	< 0.001
	Abdominal strength (%)	47.7	49.3	54.9	0.025	44.6	44.2	44.4	0.979	55.6	54.1	62.6	0.020	48.4	55.5	53.9	0.001
	Aerobic fitness (%)	47.9	55.1	53.1	0.037	51.9	50.8	53	0.592	52	49.5	62.3	< 0.001	49.2	54.4	55.4	0.006
	General flexibility (%)	46	47.7	48.3	0.685	40.0	41.1	37.6	0.209	54	53.8	53.5	0.984	42	51.8	48.2	< 0.001
	Improve muscle balance (%)	36.2	39.3	28.7	< 0.001	30.7	32.6	33.6	0.282	48.2	44.6	40.9	0.043	38.3	44.4	38.9	0.010
	Other (%)	52.5	62	40.8	< 0.001	46.8	54.8	33.3	< 0.001	54.1	58.9	40.7	< 0.001	50.1	58.5	41.7	< 0.001

Values are expressed in %;* P* < 0.05.

We observed that ‘home exercise' and ‘receive/borrow equipment’ were higher in the professional group, particularly in *very high* HDI countries (Table 1). Access to exercise facilities and spaces around housing areas was greater in *high* HDI for both amateur and professional groups. Running in a recreational park, oval, or stadium was more accessible in *very high* HDI for both amateur and professional groups. Outdoor cycling was also more accessible in *very high* HDI countries. Gymnasium access was higher in *very high* HDI and for professional athletes. Academic facilities were utilized more by professional athletes across all HDI levels. Outdoor hiking was higher in *very high* HDI in both groups.

Broadly, the purpose of training was general fitness and health, as shown in more than 80% of amateur and professional ahletes (Table 1). In terms of the purpose of training, skill/technique developments were more prevalent in *low-medium* HDI without a marked difference between amateur and professional groups. A greater emphasis in strength and power, and muscular endurance training was observed among professionals from *high* HDI countries. Abdominal strength and aerobic fitness training were more prevalent among professionals from *very high* HDI countries. General flexibility was a priority for professionals, across all HDI levels. Training for muscle balance was more pravelant in professionals (mainly *high* HDI level).

### Training practices before and during the first 100 days of lockdown

Both amateur and professional athletes (female and male) reported a decrease in training frequency (− 36% for all amateurs and 39% for all elite), duration (− 33% for all amateurs and 33% for all elite) and quantity (− 54% for all amateurs and 56% for all elite) compared to pre-lockdown (Table 2). However, no significant time effect (before vs during lockdown) and HDI interactions were observed for training frequency.

**Table 2:** Present frequency of training, training duration, quantity of training, % of intensity of training maintained in the amateur and professional groups (females and males participants) according to the level of HDI (*low-medium, high* and *very high*);

	HDI	Amateur group								Professional group							
		Female				Male				Female				Male			
		Low and medium	High	Very high	p-value	Low and medium	High	Very high	p-value	Low and medium	High	Very high	p-value	Low and medium	High	Very high	p-value
Frequency of training (times per week)	Before lockdown	^$^5.9	^$§^5.89	^$#^6.2	0.017	^$§^5.8	^$§^5.6	^$^*^#^6.2	< 0.001	^#§^7.4	*^§^7.2	^#§^7.9	< 0.001	^§^6.7	^§^6.8	*^#^7.4	< 0.001
	SD	2.2	2.2	2.3		2.1	2	2.1		2.7	2.4	2.4		2.5	2.2	2.3	
	During lockdown	^$^3.8	^$§^3.74	^$#^4		^$§^3.6	^$§^3.5	^$^*^#^3.9		^#§^4.7	*^§^4.3	^#§^5		^§^3.9	^§^4	*^#^4.5	
	SD	2.6	2.4	2.9		2.5	2.4	2.6		3.1	2.6	3.2		2.9	2.6	2.9	
	p-value for main effect of time	< 0.001			0.612^I^	< 0.001			0.140^I^	< 0.001			0.401^I^	< 0.001			0.504^I^
Session duration (min)	Before lockdown	^$§^88.1	^$§^88.6	^$^*^#^93.1	< 0.001	^$§^85.9	^$§^84.3	^$^*^#^95.5	< 0.001	97.8	100.8	99.8	0,002	^§^93.5	^§^92.9	*^#^100.1	< 0.001
	SD	27.8	27.6	28.2		26	26.8	27.1		27.8	26.4	25.9		27.5	25.9	26.2	
	During lockdown	^$#§^55	*^§^59.2	^$^*^#^64.4		^$§^55	^§^55.2	*^#^68.2		^§^64.8	^§^63	*^#^72.5		^#§^60.9	*^§^57.3	*^#^69.8	
	SD	30	27.6	29		29.3	28.1	31.1		33	29	28.8		31.3	28.2	32.3	
	p-value for main effect of time	< 0.001			0.032^I^	< 0.001			0.012^I^	< 0.001			< 0.001^I^	< 0.002			0.002^I^
Quantity of training (min per week)	Before lockdown	^$§^531.1	^$§^524.8	^$^*^#^583.3	< 0.001	^$#§^508	^$^*^§^480.8	^$^*^#^602.0	< 0.001	^§^740.3	^§^738	*^#^797.9	< 0.001	^§^645.2	^§^648.5	*^#^749.1	< 0.001
	SD	277.6	282.8	290.7		261.1	257.1	292.4		369.2	334.7	338.4		337.9	309.3	318	
	During lockdown	^$§^239.2	^$§^233.7	^$^*^#^275.6		^§^224.4	^§^218.4	^$^*^#^286.8		^§^340.6	^§^294.7	*^#^386.5		^§^273.6	^§^250.5	*^#^332.1	
	SD	242.7	210.2	267.2		226.7	220.7	261.3		331.6	254.4	321.1		292.6	243.6	295.1	
	p-value for main effect of time	< 0.001			0.554^I^	< 0.001			< 0.001^I^	< 0.001			0.130^I^	< 0.001			0.006^I^

^I^*P* < 0.05 significant interaction between time and HDI level; *P < 0.05, significantly different from *low-medium* HDI group; ^#^*P* < 0.05, significantly different from *high* HDI group; ^§^*P* < 0.05 significantly different from *very high* group.

Session duration decreased by 33% and 33% in amateurs and professional groups, respectively. Variations in session duration across time (before and during lockdown), gender, and level of performance indicated that as the HDI increased, there was a corresponding decrease in training duration (before and during lockdown) (Table 2). *Post-hoc* tests revealed that training session durations were longer in *very high* HDI when compared to *high* and *low-medium* HDI levels (before lockdown). Similarly, before lockdown, *post-hoc* tests revealed a longer session duration in *very high* HDI level (among amateur and professional) athletes when compared to *high* and *low-medium* HDI countries (Table 2).

Training frequency decreased by ~ 37% and 40% for amateurs and professional groups, respectively. *Post-hoc* tests revealed that female athletes reported a higher frequency of training both before and during lockdown in *very high* HDI countries when compared to *high* HDI countries (Table 2). Similarly, *post-hoc* tests identified that male athletes reported a higher frequency of training during lockdown in *very high* HDI countries than those in *high* HDI countries.

Overall training quantity decreased by 54% and 57% in amateur and professional groups, respectively. *Post-hoc* tests revealed that both female and male groups reported a higher training quantity before and during lockdown in *very high* HDI than *low-medium* HDI countries. *Post-hoc* tests also showed that both females and males reported higher training quantity before and during lockdown in *very high* HDI countries when compared to *high* HDI countries.

Amateur athletes reported lower training frequency and quantity when compared to professionals (Table 2). Between amateurs and professionals, no differences were observed for training duration in females from *high* HDI countries, male training duration in *high* and *very high* HDI countries, and quantity of training in males of *high* HDI countries during the enforced lockdowns (Table 2).

### Adaptive behaviours of quantity of training maintainers

Table 3 summarizes the positive and negative/adverse factors associated with quantity of training maintenance during lockdown across HDI and athlete levels (we termed “YES” group, for athletes who continued training; and “NO” group, for those who did not). In *low-medium* HDI countries, professional athletes reported a reduction in quantity of training (48% in YES group vs 55% in NO group). Sports where athletes maintained their activity included weightlifting (50% YES vs 30% NO*)*; long endurance training (51% YES vs 35% NO), and innovative training (42% YES vs 29% NO*)*. Similarly, in *high* HDI countries, professional athletes also reported reductions in quantity of training (35% YES vs 52% NO). In these *high* HDI countries, athletes were able to maintain weightlifting training (47% YES vs 37% NO), cardiovascular training (79% YES vs 59% NO), speed training (40% YES vs 23% NO), and interval training (54% YES vs 35% NO). Finally, in *very high* HDI countries, more athletes reported that they were able to maintain cardiovascular training (85% YES vs 71% NO) and weightlifting training (40% YES vs 32% NO).

**Table 3:**
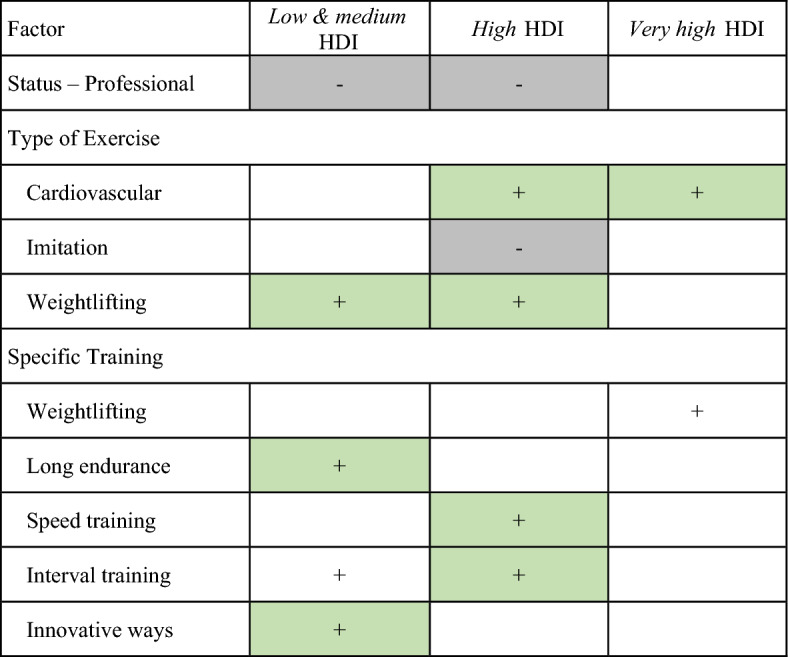
Summary of the predictive variable analysis that affected athletes during the first 100 days of the pandemic.

‘+’ indicates positive factors and ‘−’ indicates adverse factors associated to quantity of training. The predictive approach with logistic regressions and factor selection according to HDI levels used intervals for regression coefficients and p-values computed by considering the minimum and maximum values in the 20 best predictive models. We also considered the percentage of models (i.e. 100 models) and the percentage of best models (i.e. 20 models) in which the *m* predictive factors were found significant (c.f. supplemental material 1). Grey (−) or green (+) cell : the factor was found significant in 50% or more of the samples in the 100 samples AND in the 20 “best samples”.

## Discussion

**Figure 2:**
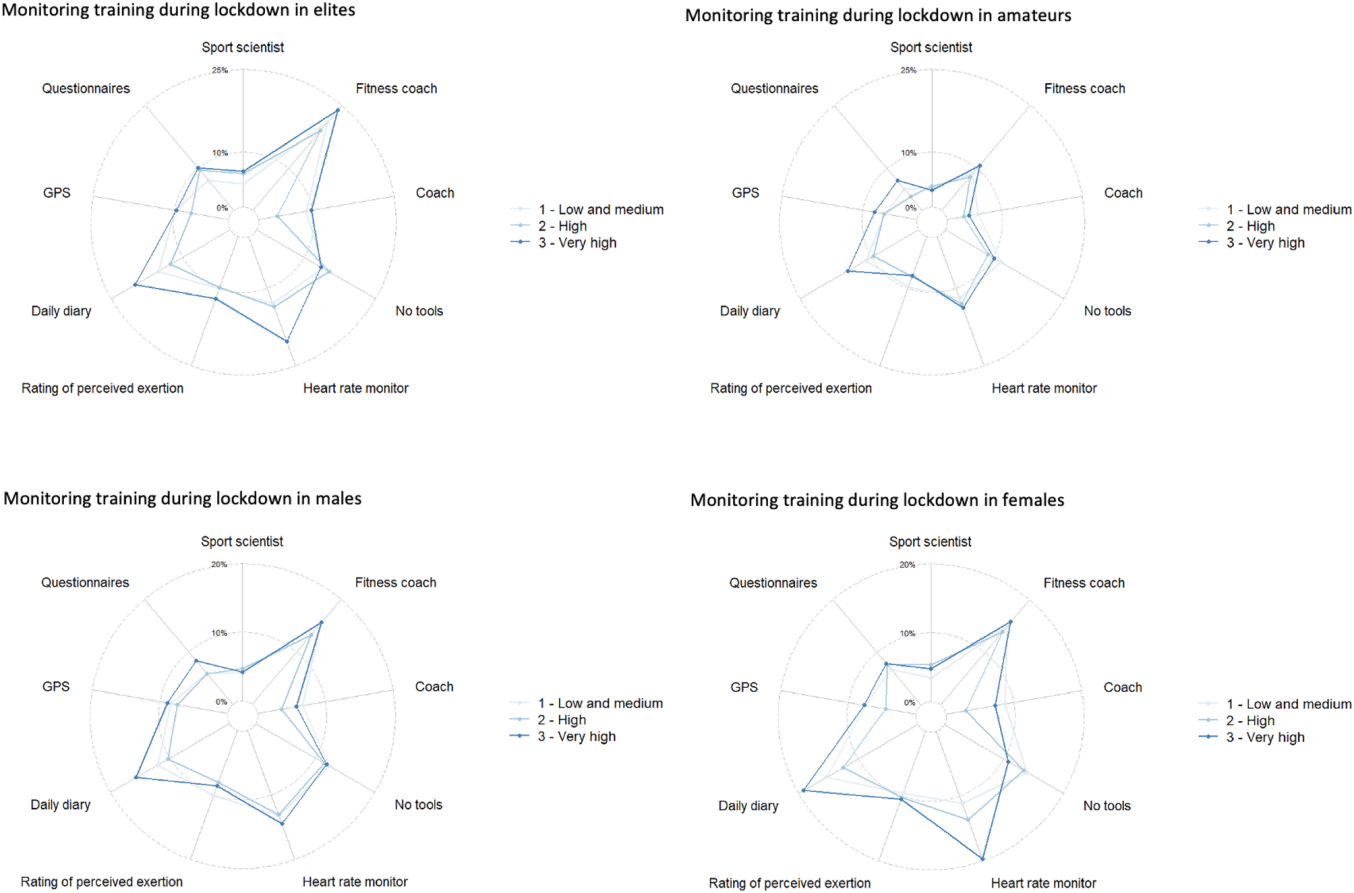
Radar plots describing the type of exercises, specific training, modes of physical recovery, monitoring of training, type of monitoring, and tools used to monitor for *low-medium* I, *high,* and *very high* HDI in female and male amateur and professional athletes during lockdown.

During the first 100 days of the COVID-19 lockdown, weekly training was dramatically impacted, reducing by 54% (287 min wk^−1^) in amateur and 57% (402 min wk^−1^) in professional athletes. This trend was consistently observed across various components of training practices (e.g., duration, frequency and quantity), irrespective of a country’s HDI and the performance level of the athletes. We found that HDI level was associated with a reduction in training duration, in both genders and across a range of performance levels during a mandated medium to high severity lockdown. Moreover, the total quantity of training (frequency x duration x intensity) was markedly reduced among both amateur and professional female athletes. Among athletes who were able to maintain their quantity of training, their training strategies were mediated by the type of exercises they chose (weightlifting or cardiovascular), and by the specific training they undertook (body weight exercises, long endurance and innovative training), Fig. 2.

### Authority rules and training

During the lockdowns, athletes adjusted their training approaches in accordance with regulations set by the authorities in their respective countries. In countries with *low-medium* HDIs, athletes were primarily limited to home-based training focused on technical skill and mobility exercises. In contrast, athletes in *high* HDI countries had more opportunities to exercise around the housing area (residences), emphasizing general fitness and health, flexibility, muscle endurance, strength and power, and muscle balance. Athletes in *very high* HDI countries were able to access even more diverse options, like running or training in parks, stadiums, cycling, and hiking in non-public facilities (i.e., permitted by local authorities). These athletes could also more often receive or borrow training equipment, and/or gain access to a gymnasium, or sport training facility during lockdown (Table 1). Accordingly, Czech et al.^17^ showed that a more stringent anti-COVID-19 policy was related to greater declines in human mobility, and that HDI levels was an important driving factor mediating the magnitude of human mobility changes during COVID-19.

During the lockdown some elite athletes reported to receive training equipment to help them train at home while under lockdown, through their clubs or sponsors^18^. Athletes from higher HDI locations generally experienced more flexible training opportunities, including access to a broader range of locations for training sessions and specialized training equipment. Both the locations and training purpose during lockdown were strongly influenced by the HDI level (Table 1). Moreover, professional athletes in *high* and *very high* HDI countries had a greater opportunity for diverse training purposes (Table 1). These data shows that there was a substantial amount of HDI-related inequality for athletes during the early COVID-19 pandemic’ lockdown that could have affected both physical, social and mental health in athletes.

### Training behaviors and performance level

We revealed a similar decline in key training variables, across duration, frequency and the product of these variables, training quantity, irrespective of HDI and performance levels (Table 2). The decline in training observed in athletes during lockdown described herein (Table 2 and Fig. 1) has been reported in the general population^19–21^ and in active people^22^ or athletes^8,9^. While individuals in Europe and North America showed some recovery, their “step count data”, in early 2022 movement remained 10% lower than global prepandemic baseline (5600 steps per day) with regional variation^20^. Some early studies neglected differences in HDI levels, potentially overestimating the influence of a country’s healthcare standard, education, and socio-economic environment^17^. Moreover, limited data are available on exercise variables such as frequency, duration, quantity and intensity during lockdowns, which are the key factors in meeting moderate to vigourous physical activity international recommendations^13^. Thus, the current study (HDI influences) adds a new dimension to the existing literature, which primarily suggests that high-level, as well as professional athletes faced minimal disruption (compared to low-level, and amateur athletes) in their training routines during lockdowns^8,9, 23^.

Between amateurs and professionals, no differences were observed for training duration in females from *high* HDI countries.

We observed that the quantity of training during lockdown was strongly dependent on the HDI level across time (before and during lockdown) in male athletes. However, no differences were observed in females regardless of HDI level (Table 2) and training status. While we observed that lockdown and HDI impacted training duration in both professional and amateur male athletes, this was not the case in amateur and professional females (Table 2). Professional female athletes in *high* HDI countries, were particularly vulnerable to reduced frequency and quantity of training. This observation may be attributable to the demand of work (mainly for amateur women), as well as family responsibilities (for amateur and professional female athletes), which might have affected their training time and motivation (Table 2)^5,24^. Moreover, differences in financial ressources and support available for professionnel women could have affected their daily life and as a consequence, their training during the lockdown. With regards to gender difference, earlier studies have also reported a notable disparity in training between genders in training, such as fewer weekly training days and less total training hours in female athletes compared to their male counterparts^25^. The challenges faced by female athletes, including those living in *high* HDI countries, highlights the need for specific considerations related to gender in future health policies and guidelines.

### Maintenance or increase of training during lockdown

Using logistic regression and factor based on HDI levels, we identified the main factors that had an impact on the training maintainance during lockdown (Table 3; supplemental material 1). In *low-medium* HDI countries, the focus of “specific training” category (Table 2) was on body weight exercises, long endurance, and innovative training. Athletes were compelled to find new ways of training, typically in isolation, and frequently through online platforms, due to lockdown measures and cessation of traditional (regular) training, which involves communications between athletes and coaches^8^. In *high* and *very high* HDI countries, the focus shifted to weightlifting or cardiovascular in the “types of exercises” category (Table 2, Fig. 2), along with speed and interval training. These choices provide evidence of adaptable training strategies during lockdown due to athletes’ training knowledge and their ability to apply the training^8^. According to a mini review, typical home training (for team-sport athletes) during lockdowns consisted of about 5 weekly sessions lasting 45 to 90 min each, and primarily focused on muscular strength and endurance^26^. These practices resulted in decreased VO_2_max, slower sprint times, and inconsistent (negative/positive) changes in countermovement jump height^26^. Other studies have also indicated that a country’s socio-economic development largely influenced training behaviors^5,20^. Combined with the level of training knowledge, athletes could explore innovative ways to train beyond body weight exercises and long endurance training, as observed in global population^6^.

Somewhat surprisingly, professional athletes reported difficulties in maintaining exercise quantity in *low-medium* and *high* HDI countries, but this was not entirely the case in *very high* HDI countries (Table 3). Unlike in *very high* HDI countries, restrictions on exercise locations and lockdown durations had a negative impact in *lower* HDI countries that might relate to the socio-economic environment of these countries^5,20^. However, research on how macroeconomic factors (e.g., HDI) affect training practices, particularly during the COVID-19 crisis, is notably lacking. In the current study, athletes demonstrated adaptability in exercise regimens based on their socioeconomic and educational backgrounds. The specific strategies adopted by athletes may be applicable to wider population at similar HDI levels, incuding individuals with diverse backgrounds and health conditions (e.g., youth, old, disability or chronic disease, pregnant women), and can inform public policies and guidelines. Tailored recommendations, including digitally-mediated training^8^, can be effective at combating inactivity across all HDI levels, despite the challenges in implementation^6^. In addition, “bubble” training camps may be implemented to allow regular training with teammates (supported by coaches and performance support staff)^27,28^.

### Limitations

In this global study, we assessed training practices using a self-reported approach (questionnaire survey) rather than direct measurements, which may be seen as a limitation when interpreting quantity of training^29^. However, given the worldwide nature of the COVID-19 pandemic and the study’s reach across 121 countries, a survey approach was deemed a suitable option. It is important to note that our focus was particularly on training practices related to exercise and sports training, omitting other forms of daily physical activity such as household tasks, gardening, among others; therefore, caution should be taken when extending these study outcomes to other population groups. While distribution of participants in each category was balanced between amateur and professional in men and women, amateurs and professional males participants of *very high* HDI were not evenly distributed and this has been taken into account in the predictive approach regarding HDI (SM1).

## Conclusions

During the first 100 days of the global COVID-19 pandemic, the training quantity decreased by 54% in amateur and 57% in professional athletes across varying HDI countries *(low-medium, high*, and *very high*). Athletes in *low-medium* HDI countries focused more on innovative training, even though female and amateur athletes experienced a substantial reduction in training quantity. Athletes from countries with a higher HDI and participation in sports competitions (i.e., professional athletes) had more opportunities to diversify training practices during lockdown. Access equipment and facilities, as well as opportunities to perform training away from household, were the primary factors influencing training practices. Factors such as lockdown rules, socioeconomic environment, and training education may have influenced the limited diversification of training and approaches, particularly in *low-medium* HDI countries. Amateur and professional athletes who maintained the training quantity during lockdown, prioritized cardiovascular and strength training, irrespective of the HDI. This study has implications for both sports and public health policies and guidelines in all countries. Educational programs tailored to gender, performance and HDI levels should offer opportunities to improve training practices during challenging situations as lockdown, or lockdown-like situations.

### Supplementary Information

Supplementary Information.

## Data Availability

All data are stored on institutional servers of the corresponding author and are available on reasonable request. All related survey questionnaires are presented in the main text or within the supplementary material.
