# Supplementary material for: Training strategies of 10,074 athletes from 121 countries based on human development index in early COVID-19 lockdown
Source: Sci Rep. 2024 Apr 17;14:8866. doi: 10.1038/s41598-024-59375-y (PMC11024144; doi:10.1038/s41598-024-59375-y)
Supplement: Supplementary file 1 — Supplementary Information. [file 41598_2024_59375_MOESM1_ESM.docx]

**Supplemental material 1: Method used to determine the most predictive factors for quantity of training maintenance**

**Method overview**

To understand what could lead participants to maintain and/or increase their quantity of training during lockdown, we introduced a binary output called quantity of training maintenance and then we processed data according to HDI levels to determine the main factors explaining the introduced binary output. For each HDI level the process, based on a predictive approach, consisted in computing logistic regressions randomly sampled (with a training and a test sample) and designed with a stepwise (backward – forward) process for factor selection. Then models were ordered according to their accuracy and relevance assessed thanks to indicators such as Akaike Information Criterion (AIC), counting the explaining factors and counting the well-classified participants.

The most predictive factors were determined by counting the models (amongst the 100 different built models and amongst the 20 best models) in which they were found significant.

**Details about the method**

The method used was similar for each HDI level. Since there are many more participants classified as “NO” in the output factor when compared with those classified as “YES”, our sample was unbalanced (around 91% and 9% respectively). Therefore, we needed to use a special process for balancing. The following steps describing the full process used were computed 100 times for each HDI level providing models with their indicators:

Randomly split data into two subsamples

- - 80% data into the “naïve training sample”
  - 20% data into the test sample

1. Balance the training sample
   - Remove the “NO” individuals detected with the Tomek’s procedure^29^
2. Randomly remove other “NO” individuals in order to get n=1.2 times more “No” individuals than “YES”
3. Design the predictive model on the balanced training dataset with the backward – forward process using the Akaike information criterion (AIC)
4. Apply the model on the test sample
5. Compute indicators for relevance and accuracy of the models
   - Counts and percentages of individuals well classified by the model
   - Counts and percentages of “YES” participants well classified by the model
   - Counts of explaining factors
   - (AIC previously computed)

The assessment of relevance and accuracy was used to select 20 predictive models, called “20 best models”.

The resulting models were used to evaluate the explaining factors through indicators as described hereafter:

1. For each predictive model, counting the number of predictive factors and then computing the resulting median value, noted
2. For each predictive factor, counting the number of models in which they were found significant
3. Consider the predictive factors which appeared the most numerous times in predictive models

The most predictive factors were evaluated by the percentage of models (i.e. 100 models) and the percentage of the “20 best models” in which they were found significant (i.e. . Explaining factors appearing in at least 50% in both the 100 models and the “20 best models” were considered as the most predictive ones. Intervals for regression coefficients and p-values showed in Table 4 were computed by considering min and max values in the “20 best models”.

Figure 3 is a schema of the process previously explained.


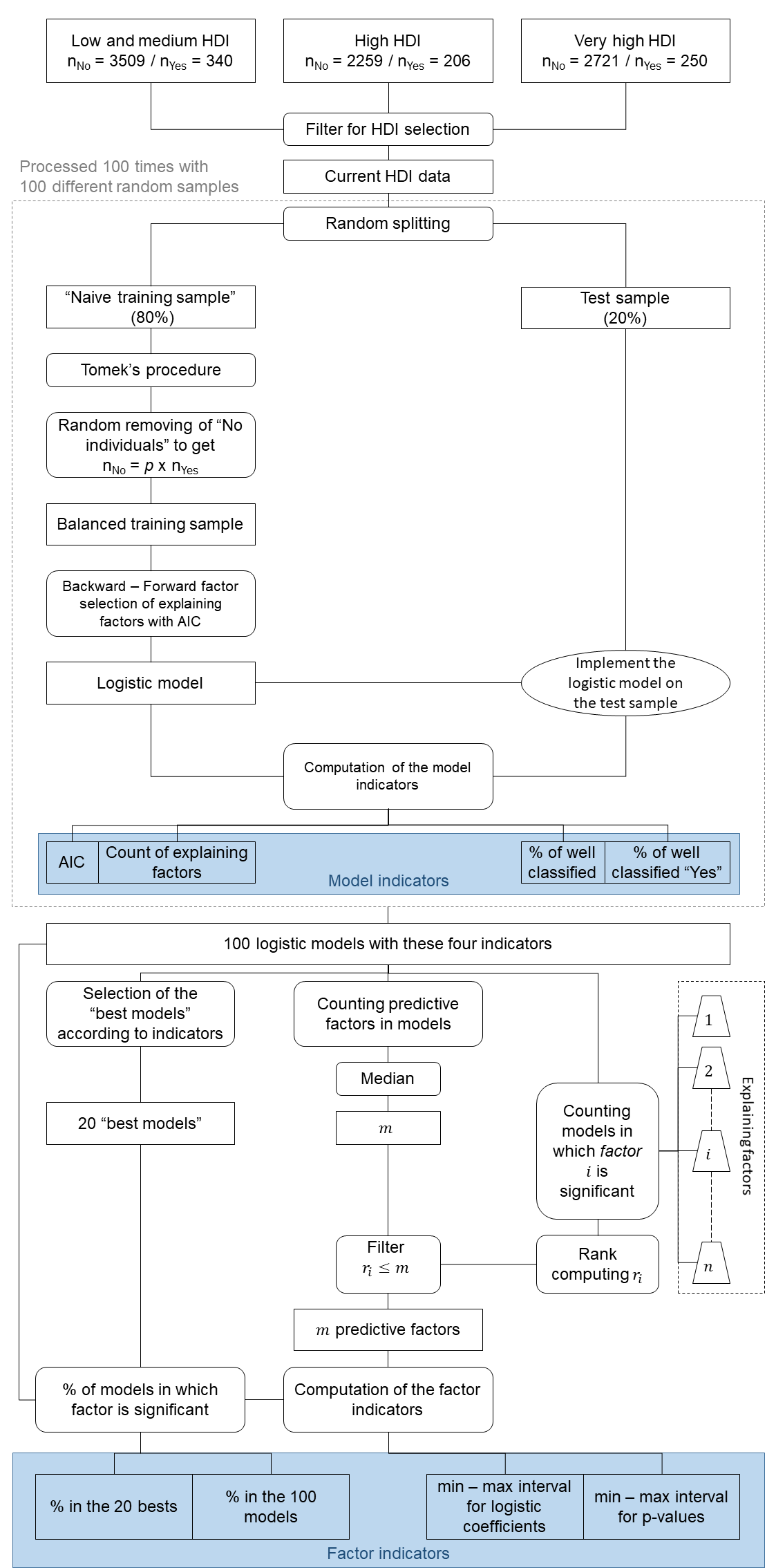


**Figure 3:** Schematic of the process used to determine the most predictive factors for quantity of training maintenance
